# A microcomputer automated circular dichroism spectropolarimeter

**DOI:** 10.1155/S1463924681000394

**Published:** 1981

**Authors:** Victor C. Zadnik, Karl H. Pearson, Robert Megargle

**Affiliations:** 1 Department of Chemistry Cleveland State University Cleveland Ohio 44115 USA; 2 Aluminum Company of America Alcoa Technical Center Alcoa Center Pa 15069 USA


### A microcomputer automated circular dichroism spectropolarimeter

**Victor C. Zadnik*, Karl H. Pearson, and Robert Megargle****
*Department of Chemistry, Cleveland State University, Cleveland, Ohio 44115, USA.*

### Introduction
Circular dichroism (CD) measurements are especially useful in conformational and structural studies, and have proved to be a valuable tool in biochemical and medical investigations. The technique is also suited to the quantitative analysis of optically active substances. The value of the tool can be improved by computer processing of results as it is useful to be able to compare one spectrum with another, subtract a background or interfering spectrum from another to highlight the differences, average several spectra, calculate the derivative of a spectrum, merge two spectra with overlapping wavelength ranges, or produce calculated optical rotatory dispersion (ORD) spectrum from the CD spectrum.

### The Cary 61 CD spectropolarimeter
The Cary 61 spectropolarimeter is designed to measure and record circular dichroism versus wavelength in the region of 185 to 800 nm [1]. Light from a xenon source and a double prism monochromator is passed through a Rochon linear polariser and then a potassium dideuterium phosphate Pockel cell, an electro-optic polariser modulated at 325 Hz. This produces an alternating beam of left and right circularly polarised light which is passed through the sample and focused on a photomultiplier tube. The measurement system determines the difference in absorbance between the left and right components and uses this difference to record circular dichroism in terms of degrees ellipticity.

Obtaining accurate spectra with the Cary 61 circular dichroism spectropolarimeter requires a knowledgeable operator and with complex spectra can be quite time consuming. There are a number of interactive controls that must be properly set to achieve optimum results. Many of the combinations of pen period and scan rate, for example, may give inaccurate spectra, especially when rapidly varying spectral features are present. In such cases, useful spectra may require several hours of operator time. Furthermore, it is frequently necessary to perform a time consuming preliminary scan to establish the optimum parameters.

Because of these factors, it was decided early in the project to automate the Cary 61 CD to incorporate extensive and versatile computer control of the instrument as well as data acquisition and data processing. This automation was achieved with a microcomputer, an interface, some alterations to the original instrument, and extensive software development.

### Computer system
The Intel 8080 based MITS Altair (TM) model 8800A microcomputer system with 48K bytes of random access memory (RAM) and 8K bytes of erasable programmable read-only memory (EPROM) was used to automate the Cary 61. The system was equipped with dual MITS model 88-DCD floppy disc drives, a Texas Instruments Silent 700 KSR (TM) teleprinter, and a Houston Instruments HI-PLOT (TM) digital plotter. Four Motorola MC6820 peripheral interface adapters (PIA) with dual 8-bit input/output channels on a MITS model 4-PIO parallel interface card were used for data acquisition and control of the Cary 61. Five of the channels were used for output and three for input.

### Interface circuitry
The schematics in Figures 1 to 5 depict a scheme allowing the computer to acquire data and to control many of the functions of the instrument. The design allows operation in the usual manual mode, a completely computer controlled mode, or with some functions operated manually and others under computer control. The design also maintains as much of the integrity of the existing instrument as possible. Whenever feasible, the interface circuits were designed to extract or insert signals at appropriate points in the instrument, without disrupting existing circuitry, so that the quality of both the instrument and the resultant data were not compromised by external sources. Many of the connections were made at two external jumper cables provided for this purpose on the left side of the instrument, and labelled J111/J112 by the manufacturer. Shaded regions in these figures are portions of the original instrument that were included to improve clarity.

An AUTO/MAN switch was added to the Cary 61 front panel and is used to enable most of the features of the interface. It is shown in Figure 1, but this signal activates circuits in the other figures as well. An LED on the Cary 61 panel lights to indicate when the instrument is in the AUTO mode.

### Scan control
Scan speed in the Cary 61 is either variable or one of three precision rates which may each be used in the 1X or 10X mode. If the computer is not able to assert control of the scan speed, either because the AUTO/MAN switch is at MAN or the FWD-OFF-REV (which has been renamed FWD-AUTO/OFF-REV) switch of the Cary 61 is not at AUTO/OFF, then the 74155 decoder in Figure 1 is disabled and the original Cary 61 Scan Speed switch causes a ground closure at one of the pins l, m, n, or e of the connector P111. This is the original scheme in the Cary 61. A ground at pins l, m, or n selects one of the precision scans and a ground at e disables the precision scan and allows the variable scan to work. When the computer can assert control, the 74155 decoder is used to select one of the precision scans or the variable scan by a ground closure similar to the original design. LED's were added around the Scan Speed switch to tell the operator which precision scan was being selected by the computer.

In precision scan, the Cary 61 supplies a pre-selected fixed voltage to J111-g (the scan servo amplifier input) from J112-g. In variable mode, this voltage is selected by a knob on the instrument. Under computer control, a signal comes from a ±5 V digital-to-analog converter (DAC) amplified to ±15 V. For computer selected precision scans, the DAC

---

**Present address: Aluminum Company of America, Alcoa Technical Center, Alcoa Center, Pa. 15069.*

***Address correspondence to this author.*

must be set correctly by the software. Since the interface was wired so the FWD-AUTO/OFF-REV switch of Cary 61 must be AUTO/OFF, there is no competition from a signal at J112-g when the computer generates one to J111-g.

Scan direction is controlled only by the polarity of the signal sent to the scan servo amplifier. The most significant bit of the DAC-198B is called the Scan Direction Control line and was also connected to close one of two relays that switches the upper or lower scan limit switch into the Cary 61 scan motor circuit. This protects the instrument in the event of a software error that might try to drive the monochromator past its limits.

A şeparate Scan On/Off Control line is used to allow the computer to turn on the scan or leave it off. If it is off, neither of the limit switches is engaged and the scan motor will not turn. Also, no signal is sent to the scan servo amplifier and the chart engage and pen controls are inactive. The Chart Control line powers the chart engage or chart disengage solenoids. A charge pulse builds up between uses and is sent through the selected solenoids when the relay changes state. In order for the circuit to work, the Cary 61 chart control must be set at OUT. The Pen Control line activates the pen solenoid to raise or lower the pen.

**Signal measurement**

The circular dichroism (CD) or dynode voltage (DV) signal, selected by the CD-OFF-DV switch on the Cary 61, is normally sent through connector J111-f to J112-f on its way to the chart recorder circuits. Relay R9 in Figure 2 was included to permit the computer the option of supplying its own signal from a 10-bit ±5 volt DAC to the chart recorder input. This permits computer generated spectra on the Cary 61 recorder. When the computer takes control of the Cary 61 recorder by activating the Remote Recorder Control line, relay R7 is also activated to turn on the pen motor. In essence, it duplicates the function of Cary 61 switch S191B.

This same DAC may also be used to supply a replacement for the normal zero suppression control of the Cary 61. Relay R8 must be activated by the Auto Zero Control line for this option. The two uses of this DAC are not conflicting. It is not necessary to offset the CD signal going to the chart recorder when that recoder is plotting computer generated signals.

The CD signal is available at J111-f if the CD-OFF-DV switch is at CD. This signal, which varies between -5 and +5 volts, is applied to channel 0 of an 8-line computer controlled multiplexer. This is the input of a typical analog-to-digital converter (ADC) data acquisition circuit that accepts ±5 volt signals and converts it to a 12-bit two's compliment value within 100 microseconds after its start conversion (STCV) input is pulsed. The A/D converter was calibrated with a precision voltage source and found to agree with expected linearity to within one count. Note in Figure 2 that when R9 is on, the computer can read the 10-bit DAC for self-test routines.

The DV signal, obtained directly from the CD/DV switch, is 0 to +10 volts. It is offset to ±5 volts by the operational amplifier circuit at the top of Figure 2 and applied to input 1

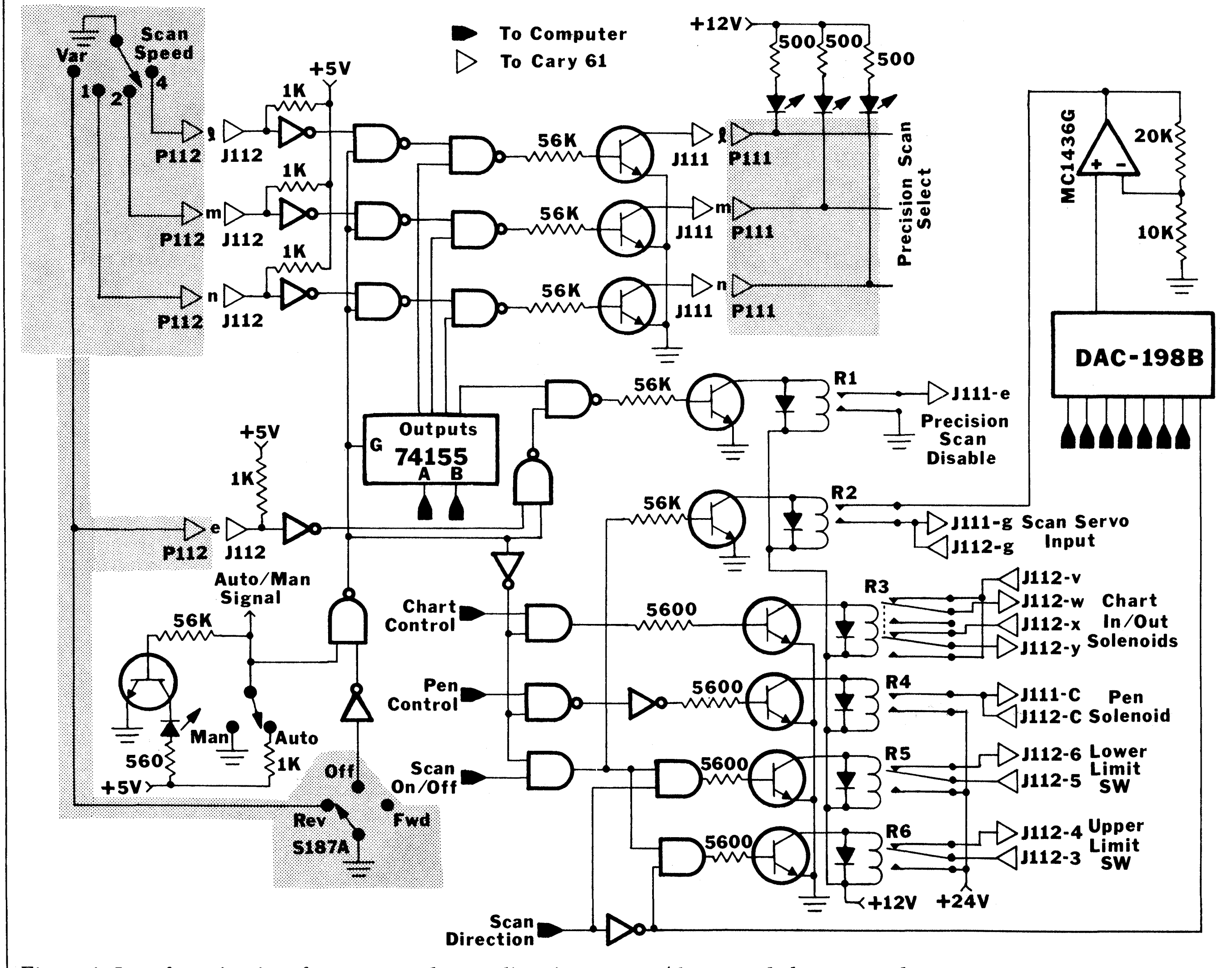

*Figure 1. Interface circuitry for scan speed, scan direction, pen up/down, and chart control.*

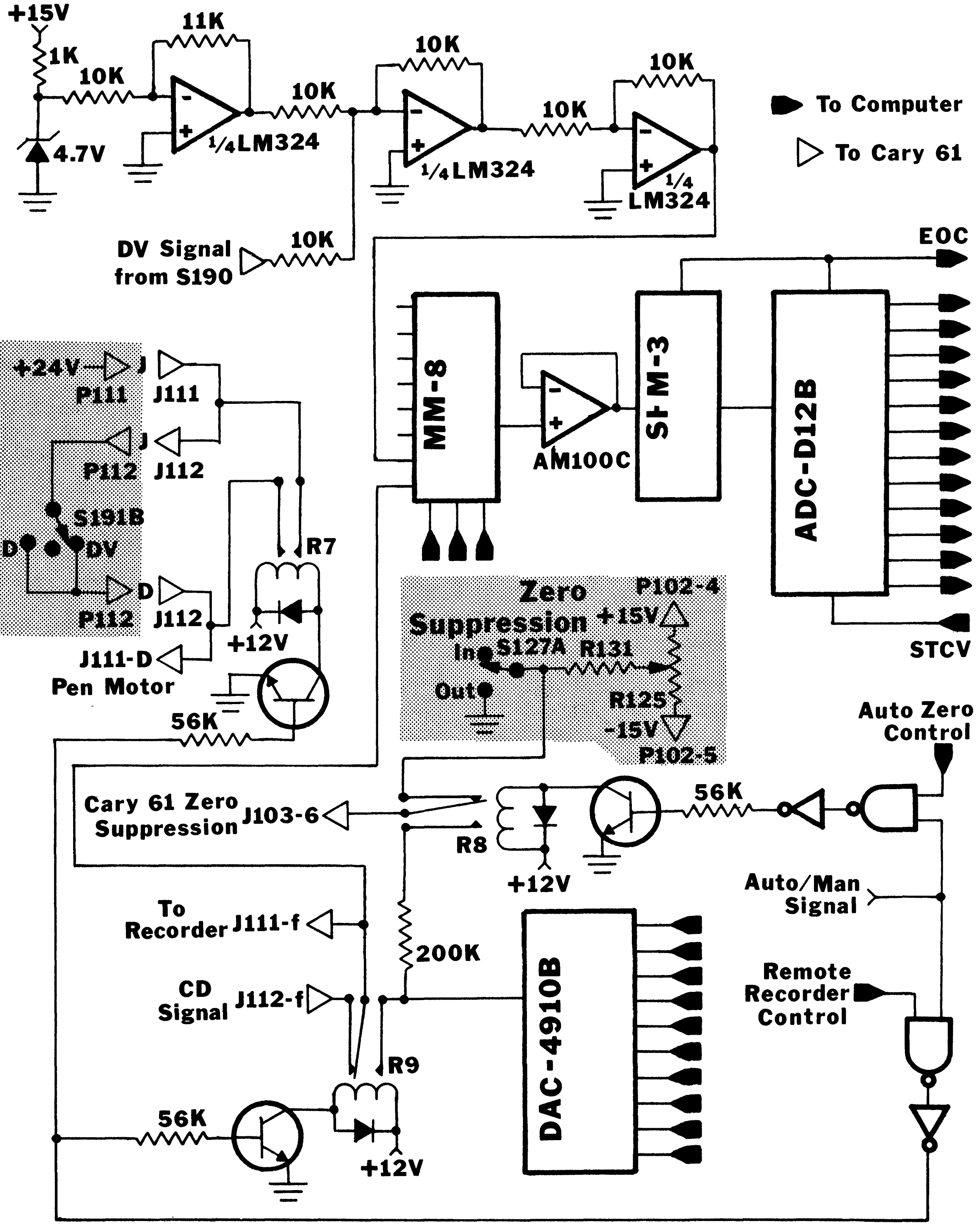

*Figure 2. Data acquisition and remote zero suppression interface.*

of the multiplexer. The DV input is only used to establish that adequate photosignal exists, and a precision measurement circuit is not necessary.

**Range and period control**

Computer control of the range function was accomplished by replacing the original rotary multipole manual switch and associated parts with a computer/manual controlled relay switching network to exactly duplicate this function. Figure 3 shows this circuit. A new manual rotary switch with one additional position labelled 'auto' was used. When auto is selected, a 3-to-8 line decoder is enabled and computer output selects the desired relay. The resistor network at the right of the figure is identical to the original instrument and was adjusted to give the same resistance readings as the original during bench testing. The new circuit was also tested for reproducibility, noise levels, and correct adjustment after installation. A series of LED indicators was added on the front panel around this switch to indicate which range the computer is using. They are connected to the relay driver transistor as shown in Figure 3.

The period switch, in Figure 4, was automated in a very similar manner. Each transistor driver activates one relay with four sets of poles. Each set is connected to a capacitor network identical to the original circuit. These 4-pole relays replace the original multi-deck rotary switch. The interconnections in the gate decoding circuit provide the functional equivalent of the original 6-position rotary switching pattern. A series of LED's are positioned around the rotary switch to show the selected position. They are driven by separate gates. There are two unneeded decoder outputs from the 74155. They are connected to select the lowest pen period.

**Wavelength measurement**

Two methods were implemented to permit the computer to determine the wavelength. A Vernitron model VGE31-407-3, 10-bit, 1-turn shaft encoder with Gray code output was mounted in a specially fabricated aluminum holder directly over the wavelength potentiometer assembly of the Cary 61. The encoder shaft was connected to the wavelength potentiometer shaft with a special coupling and vertical alignment was achieved through adjustable legs of the aluminum holder. The Gray code output of the encoder was converted to binary with the circuit shown in Figure 5. The encoder was rotated in its holder until it read zero when the Cary 61 was set at 810.6 nm. Then it was secured in the holder. The encoder was calibrated and found to have an average resolution of 0.71 nm/increment, resulting in about 900 increments over the wavelength range. With an accuracy of ±1/2 bit, this

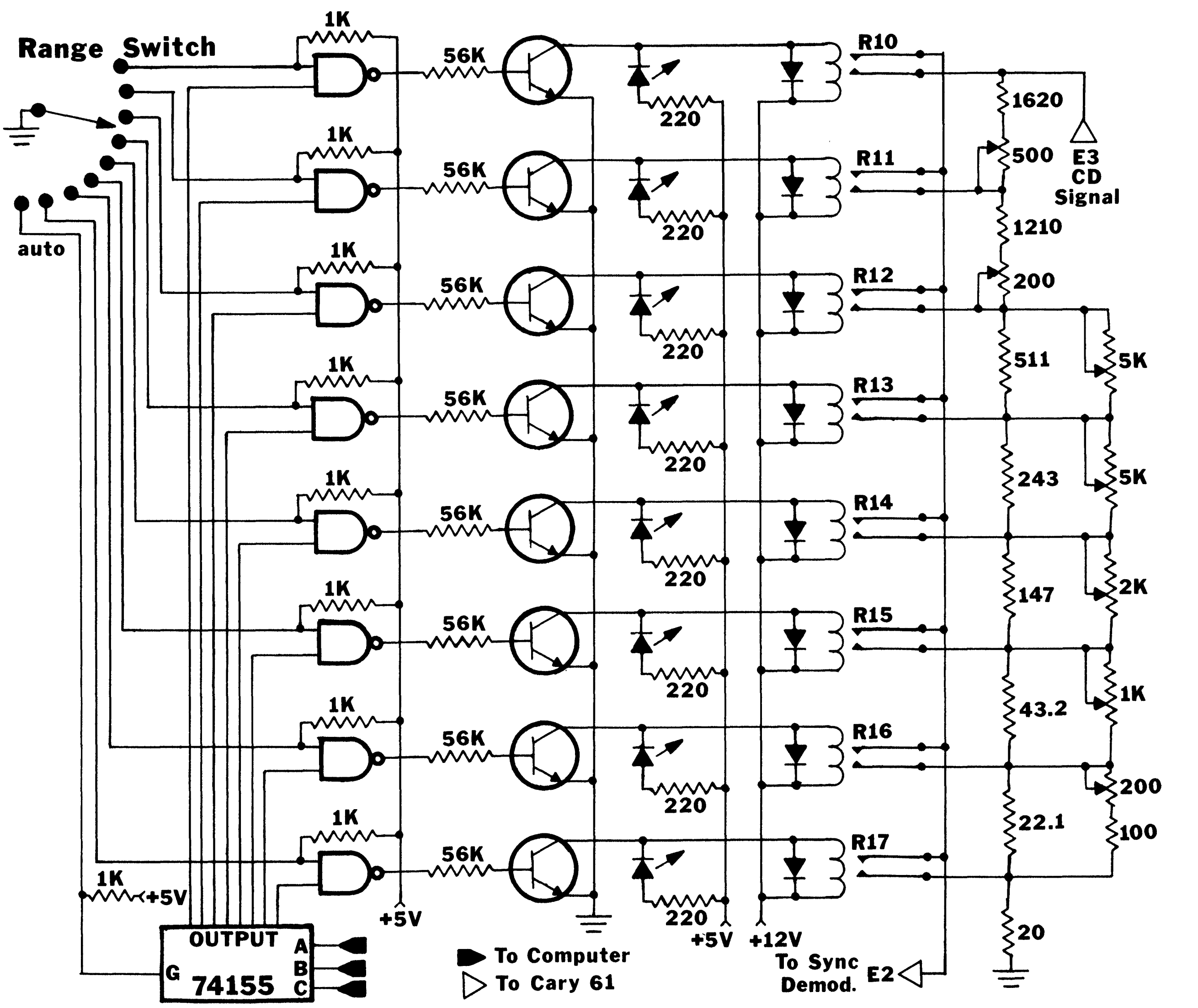

*Figure 3. Manual or computer controlled range switch.*

device could only measure the wavelength to ±0.35 nm, which is not accurate enough in most studies.

The second method used a sector wheel with photodiode detector already in the Cary 61 as a part of its tachometer precision scan circuit. This circuit produced a ±2 volt square wave at a rate of 1000 pulses/nm for a 1X scan mode and 100 pulses/nm for a 10X scan. This signal was obtained from J111-z, converted to TTL levels, and applied to an interrupt input of the computer. By keeping track of these pulses, and accurately knowing the starting wavelength, the computer could determine the present wavelength to 0.001 nm (or 0.01 nm for the 10X scan). It could only be used, however, at scan speeds between 0.03 and 0.5 nm/sec (or 0.3 to 5 nm/sec for 10X scans). At low scan speeds motor jitter caused erroneous pulses, and at high speed, the pulses were not distinctly formed.

### Software

The programs to obtain data from the Cary 61 and control the instrument were written in assembly language and stored as subroutines in about 3.5K bytes of EPROM. Application programs were written in MITS Disk Extended BASIC (Version 4.0) with linkage to the assembly language routines through user defined subroutine functions [2]. Computer controlled operation of the system incorporates a number of features which provide versatility.

The resolution and accuracy of CD results are directly related to the parameters of scan rate, pen period, and

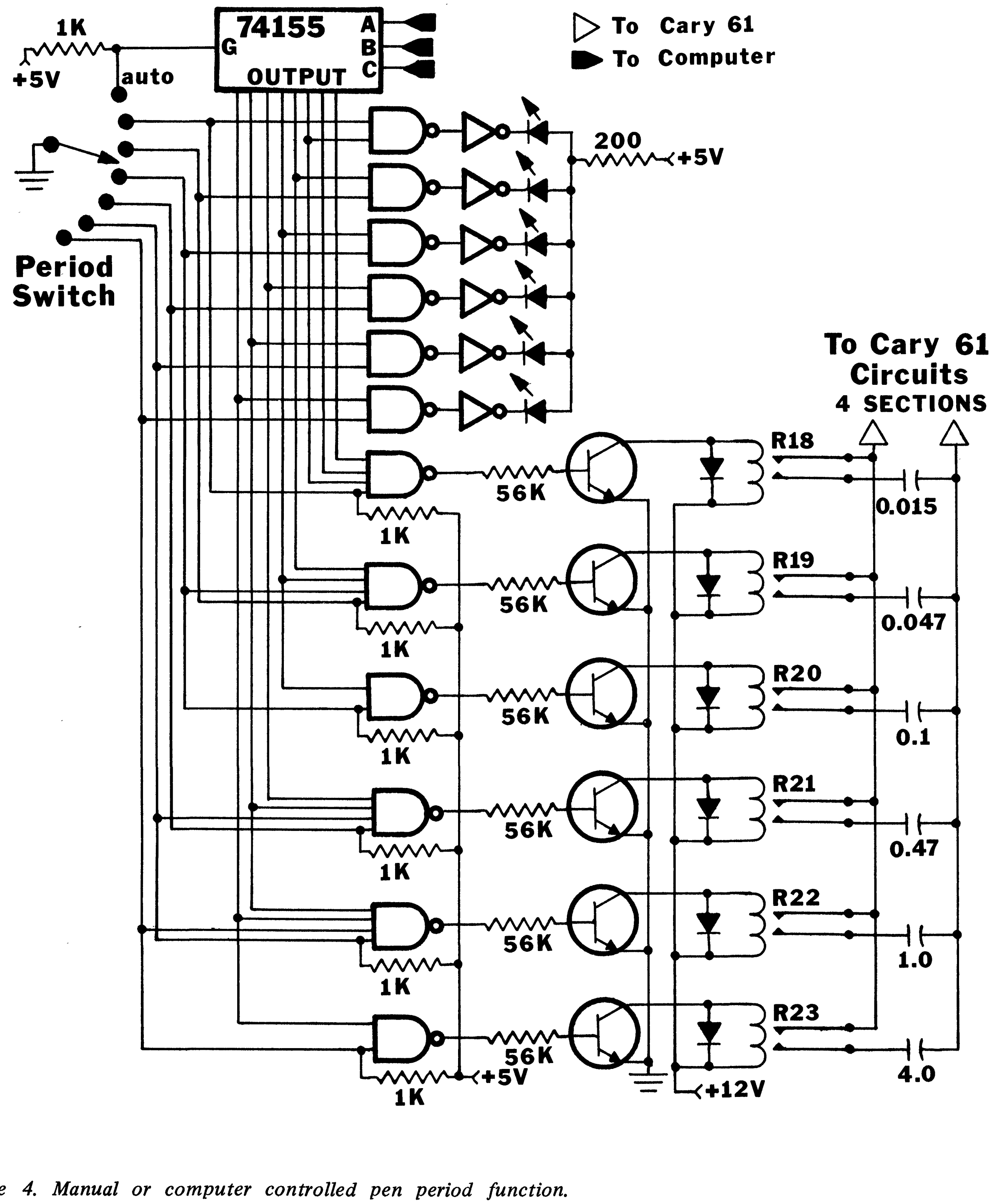

*Figure 4. Manual or computer controlled pen period function.*

measurement range. These conditions must be selected and optimised for any given spectrum on the basis of actual measurements. In manual mode, these parameters are chosen by a human operator using knowledge, training, and intuition. The computer, however, must select scan parameters using a predefined set of instructions to parallel the human process.

**Range**

The range parameter must be selected so the CD signal does not exceed the measurement limits of the circuits. If the signal goes off scale, a higher range must be set. In one algorithm, called the autoranging mode, the computer finds the minimum range that will keep the initial point of the spectrum on scale. During the scan, the computer continually monitors the CD signal and increases range when values exceed limits predefined in a range table. If the auto pen period is also selected, the period test described below is started over from the lowest period setting to locate the optimum value for each new range. In a second algorithm, called the pre-scan mode, the computer performs one fairly rapid scan at the highest range setting and computes the optimum range and zero suppression for a second high quality scan. The final spectrum is centred and uses the optimum range. A third fixed range mode was also provided where the operator defines the range setting to be used.

CD data is only valid if the sample is not too absorbing and sufficient detector energy is available. The computer verifies this by using the ADC to monitor the DV signal as well as the CD during the experiment. CD data is saved but flagged as invalid if the DV signal was above a specified value. For this instrument, a DV level above 650 volts was deemed unacceptable. The entire run was defaulted if five consecutive nm were scanned with excessive DV readings.

**Scan speed**

The optimum scan rate is dependent on the settings of pen period, slit program, slit multiplier, and the CD signal itself. For a given setting of these parameters, the maximum allowable scan rate is defined as :

$$\text{Maximum speed} = \frac{\text{Spectral bandwidth}}{\text{Pen period}} \quad (1)$$

where the spectral bandwidth is a function of the slit program [3]. A slit multiplier setting of 1 equals a spectral band-

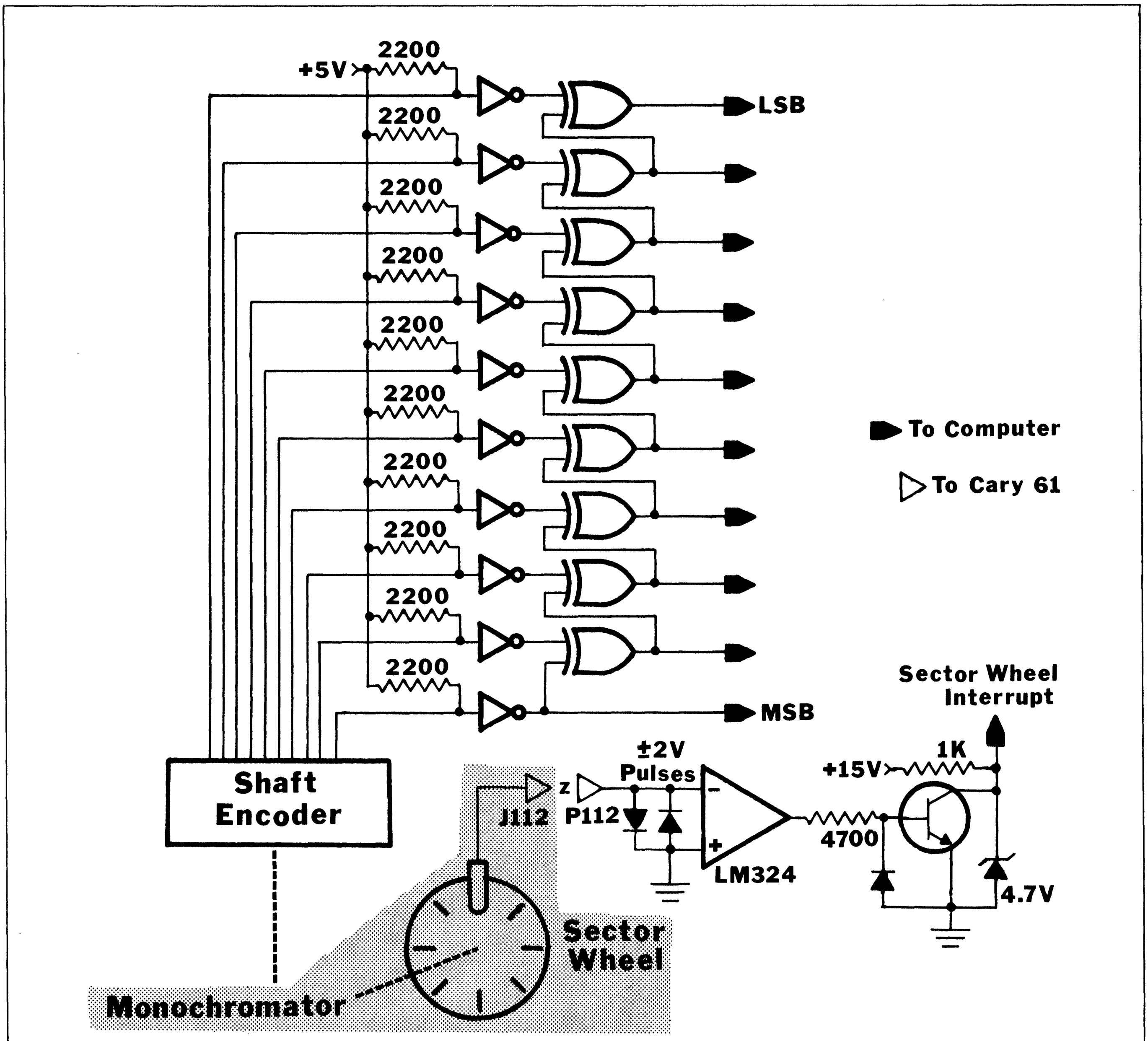

*Figure 5. Interface circuits for wavelength detection.*

width of 2 nm. All other slit multiplier settings (0.1, 0.5, 2, 10) are proportional. The maximum speed guarantees that information will not be presented to the instrument faster than the instrument response time. The optimum scan rate may be less than this maximum if the rate of change in the measured CD signal is large. The error in the measured signal can be estimated as:

$$\text{Error} = K\frac{\Delta E_{OBS}}{\Delta t} \qquad (2)$$

where K is a constant dependent on the pen period setting of the instrument, and $\frac{\Delta E_{OBS}}{\Delta t}$ is the observed signal difference between two readings spaced $\Delta t$ seconds apart. The pen period setting is the time in seconds required to achieve 98.7% of a sudden change in input. For this system, $\Delta t$ was specified as the time in seconds for a 0.1 nm increment in the scan. The constant K was computed for each pen period setting from equation 2. For example, after 3 seconds for a pen period setting of 3, the error is 1.3% of $\Delta E_{OBS}$ and $K = (3\text{ sec})\ (0.013 \times \Delta E_{OBS}\text{ volts})/(\Delta E_{OBS}\text{ volts}) = 0.039$ sec. If an acceptable error is specified, and K is known, the maximum scan rate $\Delta E/\Delta t$ can be determined. For this system, an error of two counts (or approximately 0.05% of the full scale range) was used. When auto scan rate was selected, the signal difference between successive 0.1 nm data points was continuously monitored and used to decide whether to increase or decrease the scan rate. Speeds were decreased immediately after only one large slope but ten consecutive small slopes were required before a speed increase was made. This prevented excessive speed changes when slopes were borderline. Thirteen scan rates between 0.03 and 0.5 nm/sec were implemented. The 10X scan rate feature was not supported.

### Pen period

Selection of the Cary 61 pen period depends on the amount of random noise superimposed on the measured signal. A human operator would be guided by visual observation of the recorder signal. For the computer, 0.4 nm intervals of the observed spectrum were treated as straight lines. Each interval was bounded by a measured first and last point, $P_F$ and $P_L$, respectively, and contained one centre point, $P_C$, 0.2 nm from either end. Let a noise estimate be formed as:

$$\text{Noise} = 2P_C - P_F - P_L \qquad (3)$$

If the centre point falls on the straight line connecting the ends, then Noise equals 0.0. The expression will not be zero if there is noise or the true signal vs abscissa relationship is curved. With these small intervals, the curvature effect is small. The value of Noise is compared with user specified limits, entered as a percent of full scale, and used to decide when to increase the pen period setting. Three consecutive exceptions to the limit are required for a period change. When a pen period change is made, the scan is stopped and no data is collected for 2.5 pen periods to allow the circuits to stabilise. The scan rate is changed to accommodate the new pen response. When auto pen period is selected, the computer locates the lowest period consistent with the selected noise criteria at the beginning of the scan. During the scan, the computer monitors the noise and increases the period setting if needed.

### Measurement cycles

The measurement program, written in BASIC, begins by requesting operator input. First, it asks for a file name. The results of the experiment will be stored on the disk under this name. Then the user enters the concentration and molecular weight of the sample, the path length of the cell, the temperature used, and a one line abstract that will be reproduced on the output. The program requests the starting and ending wavelengths and the wavelength interval between readings. Measurements are always taken at 0.1 nm, so that the last parameter only defines which data is stored in the disk file. The slit multiplier setting and the pen period are also entered. If auto pen period is chosen, the program asks what percent of full scale is acceptable noise. Values of 0.5 to 1.0% were typically used. Then one of the fixed ranges or the autorange feature is selected, and finally, one of the four scan modes previously discussed is chosen. The measurement program performs the scan and when it is finished, all of the information collected from the operator and the data from the run is stored in the named disk file. The file structure is shown in Figure 6. The DV character indicates valid or invalid detector energy levels for any given data point stored in the file.

### Data analysis

Separate programs are used to process and display the results. They include:

1. A difference spectrum is computed between any two spectra stored on the disc. The spectra must match each other in scan increment, wavelength region, zero suppression, and cell path length. The resultant difference spectrum is stored as a separate user named file on the disk. If a point

| | | VARIABLE TYPE |
|---|---|---|
| FILE ENTRY - 1 | ABSTRACT | ASCII |
| 2 | SCAN MODE CHARACTER | ASCII |
| 3 | STARTING WAVELENGTH (nm) | REAL |
| 4 | FINAL WAVELENGTH (nm) | " |
| 5 | SCAN INCREMENT (nm) | " |
| 6 | SLIT SETTING | " |
| 7 | PERIOD SETTING (sec.) | " |
| 8 | RANGE SETTING (deg. ellip.) | " |
| 9 | SCAN RATE (nm/sec.) | " |
| 10 | ZERO SUPP. (mdeg ellip.) | INTEGER |
| 11 | CONCENTRATION (g/ml) | REAL |
| 12 | PATH LENGTH (dm) | " |
| 13 | MOLECULAR WEIGHT | " |
| 14 | TEMPERATURE ( °C ) | " |
| 15 | CD VALUE - 1 | " |
| 16 | DV FLAG - 1 | ASCII |
| 17 | CD VALUE - 2 | REAL |
| 18 | DV FLAG - 2 | ASCII |
| | CD VALUE - N | REAL |
| | DV FLAG - N | ASCII |
| FILE ENTRY - N | END OF FILE MARK | |

*Figure 6. Disk file storage format for circular dichroism scan data.*

from either spectrum is flagged as invalid because of insufficient energy, the result in the difference is also invalid. This routine was used extensively for baseline correction.

2. A derivative spectrum is computed as the average slope between successive sets of three points. The computed slope is associated with the middle wavelength. This derivative spectrum, which is 2 points shorter than the original, is stored as a separate disk file using the same format as Figure 6 except the CD values are replaced by slope values. The dynode flag character is changed to D to valid points to represent 'derivative'.

3. A spectrum merge routine was written to combine two spectra with overlapping spectral regions into a single named file on the disk. This feature allows portions of a spectrum obtained with various cell path lengths, concentrations, and range settings to be combined, displayed, and used as a single entity. The spectra must have at least one wavelength in common, not be derivative spectra, and must have the same scan increment. The data is first converted to and stored as molecular ellipticity values in place of the observed ellipticity. The abstract, scan mode code, starting wavelength, molecular weight, and scan increment of file one is used for the heading of the resultant file. Data from file one is used up to a user defined point of overlap. Any additional data in file one is not used. Then data from file two starting from the point of overlap is copied into the resultant file, and the final wavelength for the heading of the new file is taken from file two. Existing dynode flag characters are transferred. Other parameters in the heading are not needed for molecular ellipticity and are stored as zeros.

4. A spectrum smoothing program uses a nine point polynomial least squares routine adapted from Savitzky and Golay [3]. The resultant smoothed spectrum, which is shorter by four points at both ends, is stored as a separate disk file. Dynode flag characters are ignored on input and changed to S on output to signify a 'smoothed' spectrum.

5. A routine was written to list in tabular form on the teleprinter the wavelength and observed ellipticity of any stored spectrum file. Those CD values acquired with insufficient detector energy are indicated by printing an X immediately after the observed CD value. Specific ellipticity, molecular ellipticity, $\Delta\epsilon$, and slope are computed and also printed.

6. A spectrum plot routine is used to draw titled spectra on the digital plotter. The user has the option of specifying the wavelength region to be plotted, the scale and labels to be used on each axis, and a title to be printed below the plot.

### Solutions

0.100% d-10-Camphorsulfonic acid (CSA) was prepared by dissolving 0.100 grams of 99% CSA crystals from Aldrich in water and diluting to 100 ml.

0.100 M Neodymium-R-(-)PDTA [Nd(R(-)PDTA)] was prepared by combining 1:1 stoichiometric amounts of a standardised neodymium (III) perchlorate solution with a standardised disodium dihydrogen R(-)-1,2-propylenediaminetetraacetate solution, buffered at pH 5.0 with acetic acid-sodium acetate buffer [4].

### Results

CD spectra were obtained using the Cary 61 system under constant nitrogen flush. Sample temperature was controlled with a Cary thermostated cell holder and a Haake Model KT-41 Kyrokool constant temperature circulating water bath set to maintain the cell solutions as 20.0°C.

Solution temperature in the quartz CD cells was monitored using a calibrated digital thermometer. Path lengths of the cylindrical cells were determined using a modified [5] Perkin-Elmer Model 241 polarimeter calibrated with solutions of 'Baker Ultrex' and National Bureau of Standards grade sucrose.

The Cary 61 was calibrated by both the methods of Chen and Yang [6] and Pearson *et al* [7].

CSA has a single gaussian-shaped CD maximum at 291 nm as shown in Figure 7 and the spectrum has low noise levels in the 350-230 nm region. This substance was chosen to test the microcomputer automated system. Table 1 shows the results of five repetitive scans of 0.100% CSA in a 1.00 cm cell using a range of 0.5 degrees ellipticity, a pen period of 10 seconds, -0.230 degrees zero suppression, and a scan rate of 0.2 nm/sec. Reproducibility, as indicated by the standard deviations, was less than 1 mdeg or 0.2% of full scale in all cases.

Manual and computer acquired data for 0.100% CSA in a 1.00 cm cell are compared in Table 2. All CD values are the averages of three replicate scans. The manual and single range computer controlled scans used the same instrument conditions as Table 1, except the zero offset was changed to -0.240 degrees. In the autorange experiment, all controllable instrument parameters were set by the computer. All results are in excellent agreement.

Below about 220 nm, the absorbance of the CSA solution increases dramatically and a 1.00 cm cell can no longer be used. A different path length or a different sample concentration must be employed. From 245-195 nm, the 0.100%

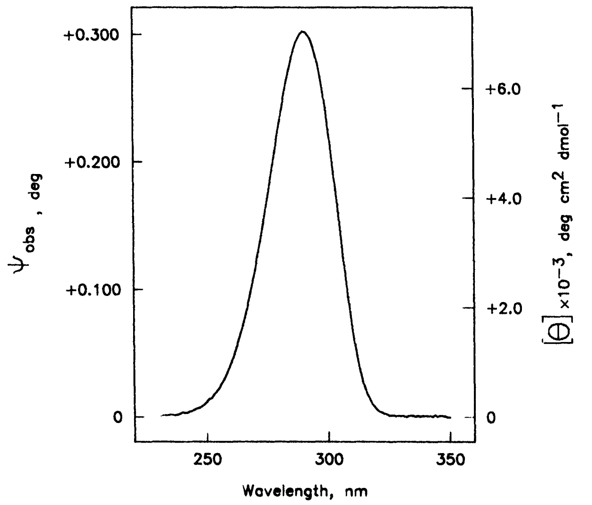

*Figure 7. Circular dichroism spectrum of d-10-camphorsulfonic acid from 230 to 350 nm. Conc = 0.100%, cell length = 1.00 cm, and t = 20.0 deg C.*

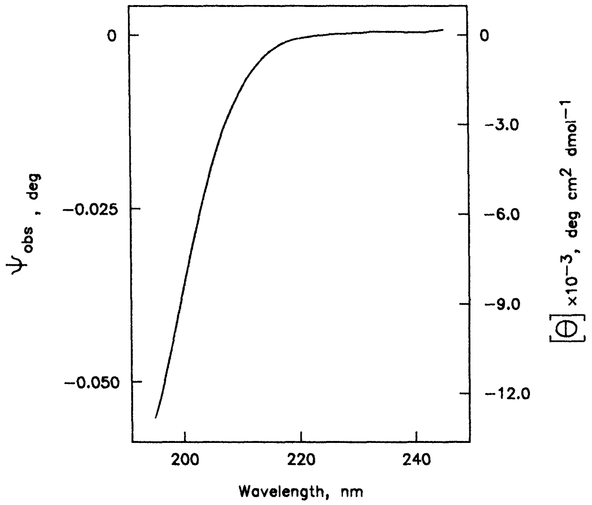

*Figure 8. Circular dichroism spectrum of d-10-camphorsulfonic acid from 195 to 245 nm. Conc = 0.100%. cell length = 1.00 mm, and t = 20.0 deg C.*

**Table 1. CD results for five computer acquired repetitive scans of CSA. Conc = 0.100 %, Cell length = 1.00 cm, Temp = 20.0 deg C**

| Wavelength (nm) | Average ellipiticity (deg) | Standard deviation (deg) |
|---|---|---|
| 320 | 0.0056 | 0.0001 |
| 315 | 0.0231 | 0.0003 |
| 320 | 0.0686 | 0.0007 |
| 305 | 0.1452 | 0.0008 |
| 300 | 0.2277 | 0.0008 |
| 295 | 0.2854 | 0.0005 |
| 290 | 0.3029 | 0.0002 |
| 285 | 0.2800 | 0.0004 |
| 280 | 0.2299 | 0.0008 |
| 275 | 0.1696 | 0.0006 |
| 270 | 0.1147 | 0.0005 |
| 265 | 0.0717 | 0.0004 |
| 260 | 0.0412 | 0.0003 |
| 255 | 0.0221 | 0.0001 |
| 250 | 0.0110 | 0.0001 |
| 245 | 0.0052 | 0.0002 |
| 240 | 0.0037 | 0.0002 |

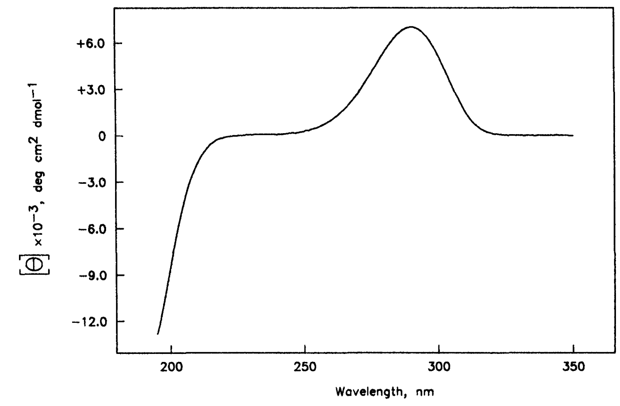

*Figure 9. Computer generated merged circular dichroism spectrum for 10-d-camphorsulfonic acid from 195 to 350 nm.*

CSA solution was run in a 1.00 mm cell at an ellipticity range of 0.1 degrees and Figure 8 shows the CD spectrum for this scan. The spectrum merge routine combines the two spectra to give the molecular ellipticity scan file for CSA shown in Figure 9. This routine eliminates the tedious calculations that would be required to achieve the same results.

The pre-scan mode was demonstrated with 0.100 M [Nd(R(-)PDTA)] solution in a 5.00 cm cell. Figure 10A shows the initial scan obtained by the computer at a measurement range of 2.0 degrees. While this spectrum shows few features visually, the ADC had sufficient resolution to determine both the most sensitive CD range and the required zero suppression. For this experiment, the computer determined that a range of 0.05 degrees with a zero suppression of -0.003 degrees was required. Figure 10B shows the final CD spectrum obtained by the computer using these parameters. Data for this experiment were obtained at 0.5 nm intervals at a pen period of 30 seconds. The scan rate was controlled by the computer. The extremely sharp f-f transitions of the [Nd(R(-)PDTA)] complex are clearly resolved showing the numerous CD maxima and minima in this spectral region [4]. This experiment required about 2 hours, most of which was unattended. To obtain a similar quality spectrum of an unknown substance without the computer may require several days and many trials.

### Discussion

This microcomputer automated CD spectropolarimeter system is versatile in obtaining, analysing, and displaying CD data. The accuracy, reproducibility, and resolution of the CD data obtained with the microcomputer is equal to or better than comparably obtained manual results. Another advantage is the reduction of time and effort made possible by the computer control features. Digitised spectra stored on the disk permits immediate analysis of results as well as examination and comparison of spectra acquired over a longer time period. The application of microcomputer techniques has produced a powerful and easy-to-use scanning CD spectropolarimeter.

**Table 2. Comparison of manual and computer acquired CD data for CSA. Conc = 0.100%. Cell length = 1.00 cm, t = 20.0 deg C**

| Wavelength (nm) | Observed ellipticity (deg) | | | | |
|---|---|---|---|---|---|
| | Manual | Computer single range | | Computer autorange | |
| | CD | CD | DIFF | CD | DIFF |
| 300 | 0.233 | 0.230 | 0.003 | 0.234 | 0.001 |
| 295 | 0.289 | 0.286 | 0.003 | 0.291 | 0.002 |
| 290 | 0.304 | 0.303 | 0.001 | 0.305 | 0.001 |
| 285 | 0.280 | 0.280 | 0 | 0.279 | 0.001 |
| 280 | 0.229 | 0.230 | 0.001 | 0.227 | 0.002 |
| 275 | 0.168 | 0.169 | 0.001 | 0.166 | 0.002 |
| 270 | 0.114 | 0.114 | 0 | 0.113 | 0.001 |
| 265 | 0.071 | 0.072 | 0.001 | 0.071 | 0 |
| 260 | 0.040 | 0.041 | 0.001 | 0.040 | 0 |
| 255 | 0.022 | 0.022 | 0 | 0.022 | 0 |
| 250 | 0.011 | 0.011 | 0 | 0.011 | 0 |

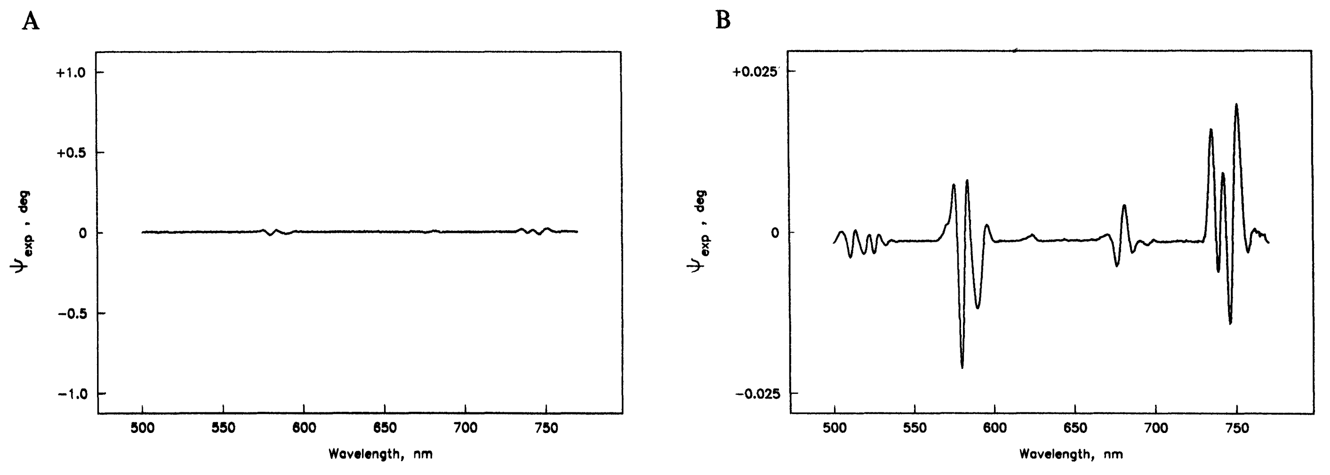

*Figure 10. Circular dichroism spectrum of [Nd(R(-) PDTA)] in which Conc = 0.100 M, cell length = 5.00 cm, and t = 20.0 deg C. (A) Initial scan at a range of 2.0 degrees ellipticity. (B) Final scan at computer selected optimum conditions.*

REFERENCES

[1] 'Cary Model 61 CD Spectropolarimeter Instruction Manual', Varian Associates, Monrovia, Calif 91016, Section 2.

[2] 'Altair 8080 BASIC Reference Manual', MITS Inc, Albuquerque, New Mexico, 1977.

[3] Savitzky, A. and Golay, M. J. E., (1978), *Anal. Chem,* **36**, 1627.

[4] Caldwell, D. L., Reinhold, P. E. and Pearson, K. H., (1970), *J Amer Chem Soc,* 92, 4554.

[5] Zadnik, V. C., Scott, J. L., Megargle, R. and Pearson, K. H., (1979), *J Auto Chem,* **1**, 206.

[6] Chen, G. C. and Yang, J. T., (1979), *Anal Letters,* **19**, 1195.

[7] Pearson, K. H., Zadnik, V. C. and Scott, J. L., (1979) *Anal Letters,* **12**, 1049.

### Notes for Contributors

### Presentation of manuscripts

Manuscripts should be typed (double-spaced) on one side of the paper only and with generous margins. The title should be brief and informative avoiding the word "new" and its synonyms. The full list of authors with their affiliations and full address(es) should appear on the title page. On a separate sheet an abstract of no more than 150 words is required. This should succinctly describe the scope of the contribution and highlight significant findings or innovations. It should be written in a style which can easily be translated into French and German.

*The Concise Oxford Dictionary* and Fowler's *Modern English Usage* (both published by Oxford University Press) should be used as the standard for spelling and grammar. Abbreviations should be limited to those generally recognised, or where a frequently occuring term is abbreviated it should, in the first instance, be explained thus "flow injection analysis (FIA) ..." and the abbreviation used thereafter. Abbreviations, for standard measures and units should follow SI recommendations. There are various publications giving guidance on the use of SI units.

References should be indicated in the text by numerals following the author's name, i.e. Skeggs [6]. On a separate sheet of paper, list all references in numerical order thus:

[6] Skeggs, L.T., *American Journal of Clinical Pathology,* 1959, **28**, 311.

Note that journal titles are given in full. Where there is more than one author, the form Foreman et al. should be used in the text but all authors should be named in the list of references. When reference is made to a chapter in a book the reference should take the following form:

[7] Malmstadt, H.V. in "Topics in Automatic Chemistry" Ed. Stockwell P.B. and Foreman J.K. 1978 Horwood, Chichester, pp. 68-70.

Only work which has been published or has been accepted for publication should be cited. Avoid the citation of documents which are subject to restricted circulation, patent literature, unpublished work and personal communications. The latter can be mentioned in the text in parenthesis.

To illustrate a paper line diagrams are preferred to photographs. Photographs should only be used when they significantly add to the discussion. Diagrams, charts and graphs should be carefully drawn in black ink on stout card or heavy quality tracing paper. Most illustrations are reduced for publication; to allow for this originals should be between 16 and 36 cm wide (the depth must not exceed 50 cm). The lettering of diagrams should be sufficiently clear to withstand reduction. Except in the case of proper names, all lettering should be in lower case print. If photographs are used they must be supplied in the form of clear, unmounted, glossy, black and white prints. "Instant" photographs are not normally acceptable. All illustrations must be identified on the reverse showing the figure number and the author's name.

Each illustration should have a fully explanitory caption. Captions should be typed together on a separate sheet of paper; they must *not* be an inseparable part of the illustration.

